# Comprehensive, atomic-level characterization of structurally characterized protein-protein interactions: the PICCOLO database

**DOI:** 10.1186/1471-2105-12-313

**Published:** 2011-07-29

**Authors:** George R Bickerton, Alicia P Higueruelo, Tom L Blundell

**Affiliations:** 1 Department of Biochemistry, University of Cambridge, Cambridge, CB2 1GA, UK; 2 Division of Biological Chemistry and Drug Discovery, College of Life Sciences, University of Dundee, Dow Street, Dundee, DD1 5EH, UK

## Abstract

**Background:**

Structural studies are increasingly providing huge amounts of information on multi-protein assemblies. Although a complete understanding of cellular processes will be dependent on an explicit characterization of the intermolecular interactions that underlie these assemblies and mediate molecular recognition, these are not well described by standard representations.

**Results:**

Here we present PICCOLO, a comprehensive relational database capturing the details of structurally characterized protein-protein interactions. Interactions are described at the level of interacting pairs of atoms, residues and polypeptide chains, with the physico-chemical nature of the interactions being characterized. Distance and angle terms are used to distinguish 12 different interaction types, including van der Waals contacts, hydrogen bonds and hydrophobic contacts. The explicit aim of PICCOLO is to underpin large-scale analyses of the properties of protein-protein interfaces. This is exemplified by an analysis of residue propensity and interface contact preferences derived from a much larger data set than previously reported. However, PICCOLO also supports detailed inspection of particular systems of interest.

**Conclusions:**

The current PICCOLO database comprises more than 260 million interacting atom pairs from 38,202 protein complexes. A web interface for the database is available at http://www-cryst.bioc.cam.ac.uk/piccolo.

## Background

Genomics provides the parts list for understanding cellular processes. However, as 70% of eukaryotic genes work through multi-protein systems [1], it is only through studying the details of these interactions that a complete picture can be gained. It is difficult to overstate the fundamental importance of protein-protein interactions as they mediate almost all cellular functions, including cell signalling, proliferation, differentiation, DNA repair and immunity. As we endeavour to gain a systems level description of these processes, it is clear that we require a greater comprehension of protein interactions, at the level both of fine details of individual molecular interactions as well as of broad principles that may be of general application. Furthermore, protein-protein interactions are being increasingly interrogated as potential drug targets [2]. Much optimism followed the discovery from alanine scanning studies that a small proportion of interface residues - the so-called "hot-spots" - contribute the majority of the free energy of binding, thereby making protein interactions amenable to modulation by small molecule ligands [3].

Structural characterization yields the most information of any experimental method, yet the details of intermolecular interactions are not described explicitly in standard representations. A range of experimental and computational techniques has been used to study protein-protein interactions, each of which provides information of a different nature, resolution and quality. Computational methods can be broadly divided into methods that identify interaction partners, those that predict interaction surfaces and those that predict the structure of the complex [4,5].

A large number of databases recording structural aspects of protein-protein interactions has been described and will be reviewed briefly here. These resources vary considerably with respect to their scope, coverage, interface definition, granularity of interface description, consideration of quaternary structure, frequency of updates and availability. Some databases consider protein-protein interactions in the form they are deposited in the PDB [6](i.e. for X-ray structures the contents of the asymmetric unit (ASU)). Others, more correctly, consider quaternary structures, identified by PQS [7], PISA [8] or from the Biological Units provided by the wwPDB (which may be either author assigned or predicted). Depending on the database the unit of interaction may be the complete polypeptide chain or the structural domain, as defined by SCOP [9] or Pfam [10]. Interfaces are typically defined either i) on the basis of changes in the solvent accessible surface area (ASA), ii) through distance based radial cut-off approaches or iii) Voronoi type procedures [11].

Recently, Meireles *et al*. described ANCHOR [12], a database of pre-computed changes in ASA undergone by each residue upon binding, as well as an estimate of the contribution to the free energy of binding, with the aim of assessing the suitability of a protein-protein interface for small molecule drug design. PISA predictions of quaternary structures were used for X-ray structures and for NMR the first deposited model was used. 3DID [13] concerns intra- and inter-molecular interactions between Pfam domains from high-resolution crystal structures and forms the basis for the InterPrets program [14]. DAPID [15] describes domain-annotated protein interactions from the PDB. Dockground [16,17] focuses on dynamic generation of non-redundant data sets of bound complexes from PDB biological units for the purposes of generating benchmarks for protein docking approaches. ICBS [18] is a specialist database of interactions mediated by interchain β-sheet formation. InterPare [19] uses radial cut-offs, difference in solvent accessibility and a Voronoi method to characterize interfaces between SCOP domains from the PDB. PIBASE [20] concerns chain-chain and domain-domain interactions derived from PDB structures and PQS assemblies. In Protein3d [21] interfaces are clustered using a sequence-order independent method that is then used to predict novel interactions through surface structural similarity [22]. PROTCOM [23] describes intermolecular interactions between PDB chains as well as intra-chain and domain-domain interfaces. In ProtBud [24], Xu *et al*. aim to facilitate comparison of the ASU, PDB biological units and PQS quaternary assemblies. Their analysis suggested that the ASU differs from PDB Biological Units in 52% of crystal structures and that PQS and PDB Biological Units disagree on 18% of entries. In SNAPPI-DB [25], Jefferson *et al*. quantified the increase in coverage of domain-domain interactions by inclusion of PQS definitions of quaternary structures. They found that the number of unique SCOP family pair interactions was increased by 13.3% by inclusion of PQS assemblies. However, when the relative orientation of the domain pairs was also considered, the PQS data increased the number of observed domain-domain interfaces by 34.5%. SCOPPI [26] interfaces are classified by the geometry of domain pairs. The resource includes multiple sequence alignments and Gene Ontology (GO) terms. SCOWLP (Structural Characterization of Water, Ligands and Proteins) [27] explicitly deals with small ligands and water molecules observed in protein interfaces.

Here we describe the establishment of PICCOLO - a comprehensive relational database of atomic level interactions from structurally characterized protein interfaces. The name PICCOLO is an approximate acronym of Protein Interaction Collection Online. In building PICCOLO our predominant focus was on providing a resource to enable large-scale analysis of global properties of protein interfaces. Thus, our requirements for a resource describing structural interactions were that a database must: i) be comprehensive, ii) have an accurate and robust interface definition, iii) describe interfaces at atomic resolution, and iv) have the capacity to remove redundancy in an appropriate manner.

PICCOLO interfaces were identified using a two-stage algorithm. An initial radial cut-off search identified atoms on different polypeptide chains that are in close proximity. A second step uses a library of residue-dependent atomic radii and a set of molecular interaction expressions comprising distance and angle criteria to flag each putative interaction from the first stage as being engaged in any of a range of defined molecular interactions. This process provides a more specific set of intermolecular atom-atom interactions than the radial cutoff method alone. Solvent accessibility calculations are then performed, allowing interface and non-interface residues to be annotated as being either buried or exposed. This enables the classification of all residues in a protein complex into one of four structural environments, depending upon whether or not they engage in interactions and their degree of solvent accessibility; residues engaging in intermolecular contacts are classed as "interface core" or "interface periphery" and all other residues simply as "core" or "exposed" as appropriate.

The PDB is highly redundant. To prevent any subsequent analysis being heavily biased, a novel pairwise clustering scheme was devised in order to generate a non-redundant set of interfaces. The compilation of a comprehensive and non-redundant set of structurally characterized interfaces enables a range of unbiased analyses of the fundamental properties of protein interfaces to be performed. Here we describe analysis of residue propensity, *i.e*. patterns of *relative *abundance of each residue type within interfaces, and residue contact preference, *i.e*. what residue-residue interactions are observed to occur more or less frequently that would be expected by chance alone.

## Methods

### Upstream data preparation

PICCOLO and its sister databases require comprehensive, up-to-date reference information regarding all solved structures currently available in the PDB. This information is housed centrally in a shared hierarchical PDB database schema, capturing annotations at the level of deposited structures, macromolecular chains and individual residues (including residue-level mapping of each PDB polypeptide to its cognate UniProt record) with automatic updates synchronized with weekly updates from the wwPDB.

Although the data found in PDB files are clearly invaluable, high-throughput processing of every structure in the repository can be hampered by the inherently heterogeneous and inconsistent nature of certain aspects of the data. A small minority of troublesome structures often require an incommensurate degree of attention to negotiate them successfully. Many of the problems stem from the fact that the PDB is not a relational database [28]. The situation has been improved somewhat by the efforts of the recent PDB remediation project [29], however many problems remain. Many of the issues can be attributed to the limitations of the particular experimental methods used to solve the structure, whereas some are due to differing assumptions made by the many thousands of different depositors over the years. Some of the issues a robust software system must handle include: crystal structures with multiple-occupancy atoms; multiple models from NMR ensembles; residue numbers with alphabetic insertion codes; inconstant presence of water molecules; inconstant presence of hydrogen atoms; absent residues in crystal structures owing to missing electron density; low-resolution structures consisting solely of Cα backbone atoms; structures containing no peptide or nucleic acid polymer residues; > 300 different non-standard amino acid residue types (naturally-occurring or engineered modifications forming part of the polypeptide backbone); low resolution structures with unassigned residue types; and lower-case or numeric chain identifiers. Unfortunately many of the standard software tools in use today do not handle these relatively common circumstances consistently. In order to isolate and avert such issues, an automated system to "sanitize" all structures on the data mirror was devised. Such pre-processing of the raw PDB data addresses the inconsistencies upstream of other processes, thereby greatly simplifying all downstream procedures and reducing the requirement for each component to perform elaborate error checking. This sanitizing process involves using the PDB module from BioPython [30] to read each structure in turn, and optionally perform a series of cleaning steps before re-writing a consistently formatted PDB file. This process ensures that only those residues that are already characterized in the database are included in the outputted PDB files, ensuring that every residue is validated and uniquely identifiable as part of a polypeptide or nucleic acid chain, thereby guaranteeing self-consistency between the cleaned PDB flat files and the database. The optional cleaning processes that can be performed include: selection of highest-occupancy atoms only; stripping of hydrogen atoms; removal of all but the first model in multi-model structures; removal of ligands; stripping of waters; and repair of the most common modified residues to their "parent" residues. Even though more than 300 different non-standard polypeptide residues can be found in the PDB, more than 90% of the total are selenomethionine (MSE), methyllysine (MLY) or hydroxyproline (HYP). Heavy selenomethionine residues are routinely synthetically engineered into proteins to help crystallographers solve the phase problem, whereas the others are more likely to be naturally occurring. The modification to their parent amino acid residue means that any such affected structures can now be appropriately handled by downstream legacy software that may otherwise break, but it does carry a small risk of incurring artefactual results (most likely false negative contact identification).

### Generation of assemblies

The atomic coordinates deposited in PDB files solved by X-ray crystallography reflect the contents of the ASU. The ASU is the minimal set of atoms which, when operated on by the crystallographic symmetry operations defined by the space group, generates the unit cell. In biological systems the space group symmetry operations are typically rotations and translations. As such, although the ASU can represent the biologically functional assembly of the protein, often it comprises multiple biological molecules or even a portion of a biological molecule. Proteins crystallize in highly non-physiological environments, at low temperatures, at artificial protein concentrations and in the presence of organic solvents and crystallization buffers, which can lead to the formation of extensive non-specific crystal packing interfaces. This has important implications for interface characterization when using ASU data. The presence of non-specific crystal contacts introduces false positive interactions. Conversely, where the ASU comprises a subset of the biologically functional oligomer, some genuine interactions will be absent, thereby introducing false negatives.

In order to circumvent these issues two versions or flavours of the PICCOLO database were built. The first flavour is derived from PDB files as they are provided by the wwPDB that for X-ray structures will represent the ASU. The ASU reflects the choice of the crystallographer in selecting a basic structural unit from which to build the crystal structure. The second flavour considers assemblies generated by the EBI's PISA resource [8], which are more likely to reflect the most biologically-relevant oligomeric assembly. As such, all subsequent analysis was performed with data derived from the PISA flavour of PICCOLO.

To generate the PISA flavour XML files containing all data pertinent to the predicted assemblies were downloaded from the PISA website, parsed and loaded into a relational database. As of June 2011 (using PISA software version 1.20) PISA comprised 164,359 assemblies in 147,439 assembly sets. Assembly sets may include more than one assembly in cases where multiple biological units are found in the ASU e.g. PDB 1c3h consists of two distinct homotrimers. The PISA procedure may identify multiple assembly sets for each PDB entry. Only the assembly set predicted to be the most stable was considered further, leaving 49,829 assemblies in 41,811 assembly sets (30.3% of the original assemblies). Often assemblies in the top-ranked set are not confidently predicted to be stable. 41,146 assemblies are labelled as "stable in solution" and this set was considered further - the remaining assemblies of lower levels of predicted stability were discarded. The relevant transformations were applied to the coordinates of the ASU of each structure. Prior to transformation any water molecules within 5Å of each polypeptide chain have their chain identifier set to that chain. A mapping is maintained between the polypeptide chain identifiers in the original ASU PDB files and the newly generated PISA-predicted assemblies. There may be more than one biomolecule for each PDB entry.

### Identification of contacts

To generate PICCOLO all PDB entries containing more than one polypeptide chain are identified. For each of these entries every unique pair of non-identical chains is examined. Therefore, for *n *chains *n(n - 1)/2 *comparisons are performed i.e. for a PDB entry with four chains A, B, C and D, six comparisons are performed (AB, AC, AD, BC, BD and CD). Note that the chain pairs are always ordered alphanumerically, preventing duplication of pairwise contacts. For each atom in the first chain of each chain pair all atoms within a fixed search radius are identified. If any of these atoms belong to the second chain, the pair is flagged as a potential inter-chain contact, the details of the two atoms are logged and the inter-atomic distance is measured. A default radius value of 6.05 Å is used, the value chosen as the maximum length of a water-mediated hydrogen bond [31]. Neighbour search algorithms such as this can be computationally expensive. However, the PDB module of BioPython implements a NeighbourSearch method using the kd-tree algorithm [32]. The *kd-tree *family of algorithms use efficient hierarchical space-partitioning data structures for recursively organizing points in a *k*-dimensional space. This gain in efficiency means that PICCOLO can be run over the entire PDB overnight on a Linux workstation of modest specification.

Definition of contacts using radial cut-offs is a commonly used approach. However, the method is considered to be sensitive but not specific, in as much as many atoms within 6.05Å of one another are unlikely to be engaged in a direct energetically significant interaction. To resolve this issue, based upon the chemical nature of the pair of atoms and the distance between them, each of the potential inter-atomic contacts is classified into a series of specific interaction types. These interaction types are listed in Table 1. In order to achieve this, each atom of the 20 canonical residues is assigned van der Waals (non-covalent) and atomic (covalent) radii as well as a series of property flags indicating the types of interactions in which they have the capacity to participate. These are described below and summarized in additional file 1: atomic_properties.xls. The values for the van der Waals and atomic radii come from intermolecular distance calculations on > 30,000 high-resolution crystal structures of small organic compounds from the Cambridge Structural Database (CSD) [33] that contain the same atomic groups as those found in proteins, such that the radius for an atom of a given element is residue-specific [34] (http://bioinfo.mbb.yale.edu/geometry/geom-mbg/data/README.htm). This set of radii has previously been used to calculate protein volumes [35]. Flags indicating those atoms that are considered hydrophobic, aromatic, cationic or anionic are set by applying SMARTs queries (SMiles ARbitrary Target Specification) (http://www.daylight.com/dayhtml/doc/theory/theory.smarts.html) to structures of the 20 canonical residues, followed by manual inspection. Each of the 20 canonical residue types also has an extra negatively ionizable OXT atom defined, to include the acidic carboxyl group when the residue is chain terminating.

**Table 1:** Interaction classification scheme.

Interaction type	Type atom *i*	Type atom *j*	Distance Criteria	Angle criteria
van der Waals	Any	any	*d(a_i_, a_j_) < vdw(a_i_)+vdw(a_j_)+0.5Å*	-

van der Waals clash	Any	any	*d(a_i_, a_j_)< vdw(a_i_)+vdw(a_j_)*	-

hydrogen bond*	hydrogen bond donor	hydrogen bond acceptor	*d(a_i_, a_j_) < 3.9Å* *d(a_h_, a_acc_) < 2.5Å*	*θ(a_don_, a_h_, a_acc_) > 90°* *θ(a_don_, a_acc_, a_acc-antecedent_) > 90°* *θ(a_h_, a_acc_, a_acc-antecedent_) > 90°*

water-mediated hydrogen bond*	hydrogen bond donor or acceptor	hydrogen bond donor or acceptor	*d(a_i_, a_j_) < 3.9Å* *d(a_h_, a_acc_) < 2.5Å*	*θ(a_don_, a_h_, a_acc_) > 90°* *θ(a_don_, a_acc_, a_acc-antecedent_) > 90°* *θ (a_h_, a_acc_, a_acc-antecedent_) > 90°*

amino-aromatic hydrogen bond*	hydrogen bond donor	amino-aromatic hydrogen bond acceptor	*d(a_i_, a_j_) < 3.9Å* *d(a_h_, a_acc_) < 2.5Å*	*θ(a_don_,a_acc_,N_aromatic-plane_) < 20°* *θ(a_don_,a_h_,N_aromatic-plane_) < 20°*

hydrophobic contact	hydrophobic	hydrophobic	*d(a_i_, a_j_) < 5.0Å*	-

Ionic	cationic	anionic	*d(a_i_, a_j_) < 6.0Å*	-

Aromatic	aromatic	aromatic	*d(a_i_, a_j_) < 6.0Å*	-

π-cation	cationic	aromatic	*d(a_i_, a_j_) < 6.0Å*	-

Disulphide	sulphur residue: cys	sulphur residue: cys	*d(a_i_, a_j_) < 2.08Å*	-

aromatic-sulphur	sulphur	aromatic	*d(a_i_, a_j_) < 5.3Å*	-

Covalent	any	any	*d(a_i_, a_j_) < cov(a_i_) + cov(a_j_)*	-

Proximal	any	any	*d(a_i_, a_j_) < 6.05Å*	-

Interactions between atom *i *and atom *j *are classified based on the atom types of *i *and *j*, the distance between them *(d) *and angle criteria *(θ*).

* indicates that the interaction is defined by HBPLUS. The following conventions have been used: *d(a_i_, a_j_) *= Euclidean distance between atoms a_i _and a_j_; *vdw(a) *= van der Waals radius of atom *a*; *cov(a) *= covalent radius of atom *a*; *θ(a_1_, a_2_, a_3_) *= angle at *a_2 _*between *a_1_-a_2 _*and *a_2_-a_3_*; *a_h _*= donated hydrogen atom; *a_don _*= hydrogen bond donor atom; *a_acc _*= hydrogen bond acceptor atom; *a_acc-antecednt _*= atom antecedent to the hydrogen bond acceptor atom and *N_aromatic-plane _*= Normal to aromatic plane. In all cases *i *and *j *are exchangeable.

Van der Waals contacts, the most common type of interaction, are assigned as those pairs of atoms whose interatomic distance is less than the sum of the van der Waals radii plus 0.5Å [21,25]. No restriction is placed on atom type. This contact definition alone is more sophisticated than many of the fixed threshold values commonly used. Van der Waals clashes are those contacts where the interatomic distance is less than the sum of the van der Waals radii. Similarly, covalent contacts are those where the interatomic distance is less than the sum of the atomic radii. The vast majority of covalent contacts are disulphides. By these definitions, covalent interactions are a subset of van der Waals clashes, which themselves are a subset of van der Waals contacts.

Unlike all other interaction types, hydrogen bonds and water-mediated hydrogen bonds are identified by an external program, HBPLUS [36]. The algorithm, developed by McDonald and Thornton, involves first positioning the hydrogen atoms, followed by calculation of the hydrogen bonds. An interaction is considered a hydrogen bond if one atom of the pair is listed as a donor and the other as an acceptor (Additional file 1), and the angles and distances formed by the relevant atoms meet the appropriate criteria (Table 1). π-electron shells of aromatic rings may also act as weak hydrogen bond acceptors [37]. In order to implement this the -R option on HBPLUS has been set to allow atoms in the aromatic rings of tyrosine, tryptophan and phenylalanine to accept these amino-aromatic hydrogen bonds.

Only in a minority of very high-resolution (< 1.0Å) crystal structures can hydrogen atoms be resolved accurately. Typically, little or no difference can be determined between carbon, nitrogen and oxygen atoms. For structures solved at resolutions greater than 1.0Å, atoms in the majority of side-chains can be uniquely identified from the electron density map, but for asparagine, glutamine and histidine, whose side-chains appear symmetrical in the electron density, certain atoms can only be identified on the basis of their local structural context and in particular their hydrogen bonds. To resolve this issue HBPLUS implements an option (-x) to explore potential hydrogen bonds that would be formed if the CD2 of histidine were actually ND1, CE1 was NE2 and the nitrogens and oxygens of the asparagine and glutamine amide groups were exchanged. Note that some atoms are capable of acting as either hydrogen-bond donors or acceptors depending on the details of their local structural context (SER OG, THR OG1, HIS ND1, CYS SG1, TRP NE1, TYR OH).

Water molecules are present in 80% of PDB structures. Although relatively rare in intra-molecular interactions, water-mediated hydrogen bonds make a significant contribution to inter-molecular interactions. Water can mediate between two hydrogen bond donors, between two acceptors or from a donor to an acceptor. One difficulty in identifying water-mediated contacts is that in many lower-resolution crystal structures water molecules may be inappropriately modelled into patches of electron density or added during refinement to improve the calculated structure factors. In this work "genuine" structured waters were distinguished by considering only those water molecules that engage in more than one hydrogen bond. Conveniently, any water molecule suggested by HBPLUS to be hydrogen bonded to two residues on different chains, by definition already meets this definition. Hydrogen bonds and water-mediated hydrogen bonds are further sub-classified as being between either two main-chain atoms, two side-chain atoms or between main-chain and side-chain.

Hydrophobic interactions are those where both atoms are labelled as hydrophobic and the inter-atomic distance is less than 5Å [38]. With respect to ionic interactions, the formally correct method of calculating the electrostatic interaction for two point charges would be to use quantum chemical methods to solve the Coulomb equation separately for each nucleus, but this is somewhat impractical for large biological systems. A simpler approach is to consider only the formal charges on the protein (whether an electron has been lost or gained). Carboxyl groups are deprotonated and carry a negative charge delocalized over the two oxygen atoms, while amino groups are protonated and carry a positive charge delocalized over the three hydrogen atoms. The protonation state of amino acid residues in free solution at pH7 can be determined from model pKa values defined for each residue. However, the protonation state of ionizable residues in the folded protein depends also on the local structure environment, including exposure to solvent, proximity to other titratable groups or permanent charges in the protein. Methods that take these factors into account [39-41] are again not practical to run at large scale, so the solution pKa values are used for ionizable residues and pH7 is assumed. The distance threshold was taken from Barlow and Thornton [42].

Aromatic interactions are defined when two criteria are met. When a pair of aromatic atoms is within the appropriate distance threshold then the centroids of the two parent planar ring systems are calculated. If the centroids are also within the distance threshold, then the contact is considered aromatic. In generating PICCOLO's sister database, CREDO [43], a procedure was devised to sub-classify aromatic contacts as being "face-to-face", "edge-to-face" or "displaced edge to face" and the same procedure was used in this work. To achieve this, for each pair of atoms involved in an aromatic contact, the normals of the two parent planar ring systems are calculated using Newell's method [44]. The dihedral angle between the two planes is defined as the angle between the normals. The displacement angle is defined as the angle between the normal of the first ring and the vector between the two ring centroids. The aromatic interaction is classified as "edge to face" where the dihedral angle is greater than 30°. Dihedral angles less than or equal to 30° are classified as "face to face" where the displacement angle is less than or equal to 20° and "displaced face to face" otherwise.

π-cation interactions are defined when a cationic atom and an aromatic atom approach within 6.0Å threshold of one another [45]. Disulphide bonds are those where two sulphur atoms from cysteine residues approach within 2.08Å [46]. Aromatic-sulphur interactions are those where an aromatic atom approaches within 5.3Å of a sulphur atom [47].

Even though these interaction definitions are not rigorous, they are each precedented, robust and rapid to calculate. Note that an exclusive classification of inter-atomic interactions would require artificial prioritization of one interaction type above another. In this work, interactions are classified equivocally so each atom pair can simultaneously exhibit the character of more than one interaction type. Each atom-pair can therefore be thought of as being represented as a binary interaction fingerprint. This results in overlaps between for example, van der Waals contacts and shorter hydrogen bonds, hydrogen bonds and shorter ionic interactions and hydrophobic and aromatic interactions. Such deliberate ambiguity arguably reflects the somewhat amorphous nature of molecular interactions. All atom pairs within the original 6.05Å distance threshold of one another are only considered as being in contact with one another if one of the above criteria are met (i.e. the logical "OR" of all interaction types). Atom pairs not meeting any of these criteria are still stored in PICCOLO as being proximal to one another, but are in general not considered in any further analyses.

### Solvent accessibility

Whilst solvent accessibility can be used to identify residues engaged in interactions, here we use solvent accessibility to annotate residues already identified by the interaction fingerprint radial cut-off method described above. The solvent accessible surface area (ASA) of a protein molecule, measured in Å^2^, can be calculated from the atomic coordinates by the program NACCESS [48] implementing the method first described by Lee and Richards [49]. To calculate the surface area that becomes buried when two molecules associate three separate calculations are performed. First the ASA of chain A and chain B are calculated separately, followed by the ASA of the A-B complex. The size of the protein-protein interface (ΔASA) is then given by:

Equation 1:

Relative accessibilities can also be calculated by expressing the accessible surface of each residue *X *relative to that observed in an Alanine-*X*-Alanine tripeptide. Absolute ASA data are calculated and stored in PICCOLO for each pairwise interaction at the level of individual amino acid residues and complete polypeptide chains. Relative accessibilities are stored at the level of individual residues. Accessibility data are stored for all residues in protein complexes - not just those residues mediating interactions.

### Filtering and clustering

The PDB is inherently redundant, with the same protein often solved multiple times under different experimental conditions, with different ligands, in different conformations and so forth, and the same is true of protein-protein complexes. Any analysis of interface properties that ignores such biases is likely to be skewed by over-represented systems. Therefore, before interface properties are analysed the data sets were filtered and clustered to provide a reliable non-redundant set. The procedure of applying PISA-derived rotation-translation matrices to generate biological assemblies removes artefactual non-specific crystal packing interfaces. Despite this a small number of insignificant interfaces remain in the PISA-derived assemblies. These typically comprise only a handful of residues, and manual examination reveals they are almost exclusively due to peripheral contacts of non-neighbouring chains in high order multiprotein systems. These were filtered out by removing those interfaces where the product of the number of residues from each side was less than or equal to 25 (R*_i _*× R*_j _*≤ 25). No chain length filtering criteria were applied prior to generation of PICCOLO. This was a deliberate choice; interactions of proteins with small peptides are of interest for small molecule drug design and when considering the effects of mutations on protein function. However, for the purposes of systematically deriving properties of protein interfaces, our primary interest was the interaction surfaces of globular proteins. Small peptidic polypeptide chains of less than 15 valid amino acid residues were therefore removed for this analysis. Collectively these filters remove 28,152 interfaces or 21.6% of the original 130,336 interfaces (data corresponds to an earlier version of PICCOLO).

Typical procedures to deal with redundant data involve performing a cluster analysis whereby the objects are partitioned into subsets such that the data in each agglomerated subset are co-proximal, as defined by a particular distance measure. Selection of one representative from each subset provides a non-redundant set. However, identifying a non-redundant set from a *pairwise *set of proteins, such as that in PICCOLO is not so straightforward. Any upstream sequence-based clustering of PDB polypeptides cannot be performed, as two structures with identical sequences may exist in different states: one may be complexed and the other bound; and even if both are bound, they may be bound to different partners; and even if both bind the same partner there is no guarantee the interaction surface or mode of interaction will be consistent.

To deal with these issues the following clustering procedure was devised. All interfaces in PICCOLO are first grouped by the unique ordered combination of UniProt[50] identifiers of both component proteins. Then within these UniProt pair clusters, each cluster member pair is compared to all other cluster member pairs and the overlap of unique UniProt residue numberings for both constituents is assessed reciprocally. If *both *sides of the interface share more than 75% of unique residue positions in common with another pairwise interaction then the interfaces are co-clustered. 75% was chosen as a sparsely populated region that gave good separation of some manually selected test cases. In order to choose representatives to form the non-redundant set, rather than simply choose an arbitrary member of each cluster, the representative complex for each cluster is chosen as the structure of the highest quality. Structure quality is quantified by using an empirical metric based on the structure's resolution, R-factor and number of absent internal residues, (with the resolution dominating):

Equation 2:

where *M *is the proportion of missing residues. This score is based on the score used by Chandonia *et al*. in deriving the ASTRAL compendium [51,52]. Note that the pairwise clustering process results in a non-redundant set of interfaces, not oligomeric assemblies.

### Residue propensity

Previous studies on residue propensities in protein-protein interfaces have drawn somewhat contradictory conclusions [53-57]. However, much of the disparity can be attributed to differences in data sets, interface definition, source of background frequency data and approaches to partitioning interaction types. Importantly many studies do not distinguish between anatomical regions of the interface. In this study the interface core and periphery are distinguished based on solvent accessibility. Residues identified in PICCOLO as engaging in interactions are classified as Interface Core if their relative sidechain solvent accessibility is less than 7% and Interface Periphery otherwise. Non-interacting residues are classified as Core and Exposed using the same threshold. Here, background residue frequency (*B_i_*) is defined, independently of structural environment, as follows for each residue type *i*:

Equation 3:

where *F_i _*is the count of each residues type calculated using all residues found in PICCOLO structures, not just interface residues. The environment-dependent residue frequency (*E_ei_*) is defined as follows:

Equation 4:

where *F_ei _*is the count of each residues type *i *in each structural environment *e*. The normalized environment-dependent propensity (*R_ei_*) is then the ratio of the environment-dependent frequency (*E_ei_*) to the background frequency (*B_i_*):

Equation 5:

### Contact pairing preferences

The frequency of pairwise residue interactions (*P_ij_*) can be derived for the PICCOLO-derived non-redundant set:

Equation 6:

where *C_ij _*represents the number of times residue type *i *is observed engaging in contacts across the interface with residue type *j*. The individual frequencies (*W_i_*) reflect the amino acid composition of each residue type *i *is be defined as:

Equation 7:

where *U_i _*represents the number of residues engaged in contacts. If interfacial amino acid residues exhibit no preference as to which residues they contact across the interface, the expected frequency of any particular residue-pair interaction would be simply the product of the two individual residue frequencies (*W_i _× W_j_*). Any such interaction preference can be quantified by calculating the log odds ratio of the *observed *interaction frequency to the *expected *interaction frequency:

Equation 8:

This measure is commonly used [58] but it does not take into account differing residue sizes (intuitively larger residues have greater surface area and therefore greater opportunity to interact with one another). Glaser [59] used residue volume data to normalize the observed frequencies. In this study we normalize the expected frequency using ASA data for each residue from NACCESS [48]. Thus, the propensity of residue-residue contacts, *L(i, j)*, is defined in Equation 8 above, but with *W_i _*replaced as follows:

Equation 9:

## Results

### Database summary statistics

A summary of the number of data points in both flavours of PICCOLO is shown in Table 2.

**Table 2:** The number of data points in the two flavours of the PICCOLO database.

	PDB Structures	PISA Quaternary Structures
PDBs (Assemblies)	38,202	36,762 (45,385)

Chains	141,133	157,166

Chain pairs	164,734	203,884

Residues	9,065,778	12,497,274

Residue pairs	14,618,400	20,450,685

Atoms	49,216,255	68,597,408

Atom pairs	184,639,194	260,224,802

### Non redundant set

The non-redundant set used to generate the residue propensities and contact preferences was derived from an earlier version of PICCOLO and comprises 14,658 interfaces.

### Residue propensity

**Figure 1:**
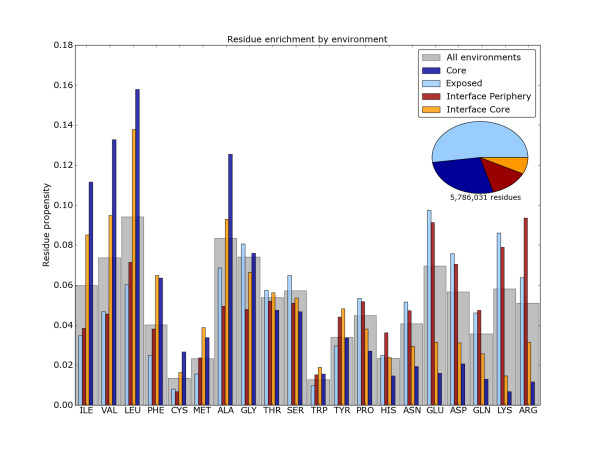
**Residue propensities for protein-protein interfaces**. The propensity of each of the 20 canonical residues for the four different structural environments suggests a highly environment-dependent distribution. Grey bars indicate the overall observed frequency across all environments (*B_i _*in Equation 3). Coloured bars indicate the environment dependent residue frequency (*E_ei _*in Equation 4). Coloured bars higher than their respective gray bars indicate the normalized environment-dependent propensity (*R_ei _*in Equation 5) is greater than 1. Residues are ordered by decreasing hydrophobicity from left to right. Inset pie chart indicates the underlying proportion of each of the four residue environments.

Figure 1 shows the residue propensities of each of the twenty standard residues for each of the four structural environments. The residues are ordered by decreasing hydropathy [60]. One overall trend is that the hydrophobic residues (Ile, Val, Leu, Phe, Met and Ala) are enriched in the protein core and interface core and conversely are depleted in the exposed surface and the interface periphery. While most of these residues are relatively enriched in the protein core than the interface core, phenylalanine is as prevalent in the interface core and not significantly depleted in the interface periphery. The polar and ionizable residues (Asp, Gln, Asn, Glu, Lys and Arg) exhibit reciprocal behaviour: they are significantly enriched on the surface and the interface periphery. Lysine is highly disfavoured in the protein core and interface core.

For the majority of residues the propensity for the interface core and periphery is intermediate between that of the protein core and the exposed surface, with the propensity pattern for the interface periphery being most similar to that of the exposed protein surface and that of the interface core most similar to that of the protein core. The exceptions to this scheme are methionine, glycine, alanine, histidine, tryptophan, tyrosine and arginine. Of these, alanine and glycine, the two smallest residues, are disfavoured at the interface periphery. Histidine and arginine, two positively charged residues, are favoured at the periphery - in fact this is the structural environment in which these residues are most enriched. Arginine is capable of multiple types of favourable interactions: it can simultaneously form up to five hydrogen bonds and an ionic salt-bridge with the positive charge carried on its guanidinium motif. Tryptophan, tyrosine and methionine, three large, hydrophobic residues that can engage in a range of interactions, are all favoured at the interface core, corresponding with the observations of Ofran and Rost [55]. The enrichment of aromatic tyrosine may be explained by its contribution to the hydrophobic effect without a large entropic penalty due to the side chain having few rotatable bonds as well as the hydrogen bonding capacity of its 4-hydroxyl group. Tryptophan has a very large aromatic side chain that can mediate aromatic π-interactions, act as a hydrogen bond donor, as well as form extensive hydrophobic contacts.

### Contact preferences

**Figure 2:**
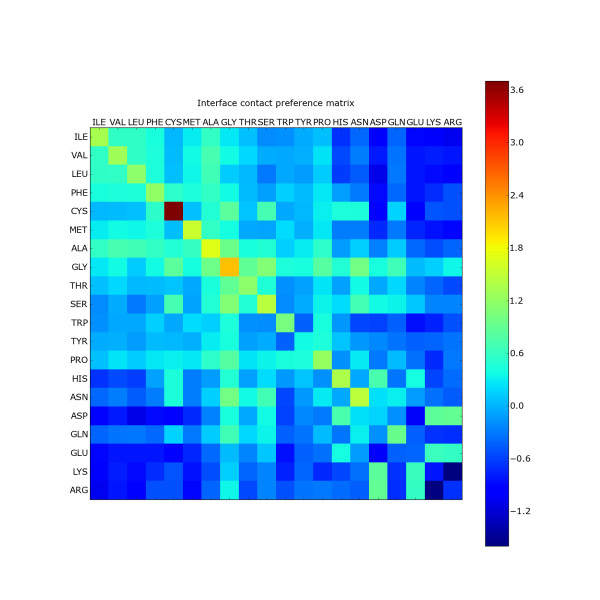
**Contact preference matrix for intermolecular residue-residue interactions**. Colours represent the log ratio of the solvent accessibility normalized observed to expected residue frequencies, L(*i, j*).

A series of matrices used in the derivation of the contact preference matrix are shown in additional files 2a, 2b and 2c, with Figure 2 showing the final contact preference matrix - the log ratio of the observed to ASA-normalized expected contacts. The progression is shown to enable assessment of the contribution of the different terms to the final contact preference matrix. Additional file 2a shows the raw observed contact matrix where leucine-leucine contacts dominate, however this is largely due to the high abundance of leucine in general. The expected contact matrix is shown in additional file 2b to illustrate the impact of residue abundance. As described in the Methods, to generate the final contact preference matrix, the expected contact matrix was normalized by the ASA of each residue. The pairwise ASA data (independent of interface contacts and residue frequencies) are shown in additional file 2c. The final contact preference matrix reveals some interesting patterns consistent with previously published studies [53,55,57,58], summarizing much of what is already established regarding macromolecular interactions - hydrophobic interactions, salt bridges and disulphide bonds are all important in protein-protein interactions. Hydrophobic residues favour other hydrophobic residues and disfavour the charged and polar residues, as would be expected from desolvation behind the hydrophobic effect. Glycine is universally favoured, most probably due to its high conformational versatility. Proline shows a preference for hydrophobic and aromatic residues - indeed it has been suggested that the interaction between a proline ring and an aromatic ring resembles the interaction between two aromatic rings [57,59]. Residues of opposing charge favour one another (arginine, lysine and histidine versus glutamate and aspartate) enabling electrostatic complementarity to be established. Like charge interactions are predictably disfavoured for the glutamate and aspartate residues carrying a negative charge (although aspartate pairs are observed near their expected rate). Interactions between residues carrying positive charge are similarly disfavoured, with arginine-arginine, lysine-lysine and arginine-lysine pairs amongst the most disfavoured. However, histidine-histidine pairs are favoured. Examination of the atomic interaction details stored in the atom pairs table revealed that a range of interactions types contribute to the histidine-histidine result, including aromatic, van der Waals, π-cation and hydrogen bonding interactions. The diagonal of the matrix is generally favoured (except for lysine pairs, arginine and glutamate pairs), likely due to the preponderance of self-interacting residues from homodimers with a 2-fold symmetry axis. The most preferred contact pairs are cysteine-cysteine followed by glycine-glycine, alanine-alanine, asparagine-asparagine and methionone- methionone. The disulphide capacity unique to cysteines plays a critical role in stabilization of small, secreted proteins. Methionine-methionine pairs are dominated by hydrophobic interactions. The tryptophan-tryptophan pairwise interactions have contributions from van der Waals and hydrophobic contacts but are dominated by edge-to-face type aromatic interactions. These results indicate some differences to those presented by Glaser [59], who found arginine-tryptophan to be the most favoured pair, which is marginally disfavoured here. However that study used a different interface definition and, as indicated in the Methods, they use residue volumes to normalize the expected interactions.

## Discussion

PICCOLO explicitly describes protein molecular interactions that are not captured in standard protein structural data representations. We believe that PICCOLO has several key features not present in any single previously published resource. These include: i) the fundamental nature of protein-protein interactions is described at maximal resolution; ii) the detailed molecular interaction terms help provide a more specific interface definition than is possible using standard radial cutoff approaches as well as providing a richer annotation of the observed interactions and iii) comprehensive coverage of structurally characterised protein complexes (both ASU and quaternary assemblies) with automated monthly updates ensures maximal data availability. These features make PICCOLO a valuable resource for researchers interested in individual systems or general properties of interfaces.

Here we have exemplified the value of PICCOLO as a platform for probing properties of protein-protein interfaces, by performing an analysis of residue propensity and residue contact preference. Aside from its value in aiding the understanding of the principles underlying molecular recognition, the residue contact preference has potential application as a source of restraints for protein-protein docking scoring functions.

PICCOLO has also been used to pursue a variety of other questions. These include *i) *the likely impact of non-synonymous Single Nucleotide Polymorphisms (nsSNPs) on molecular interactions [61,62], *ii) *the degree to which protein-protein interactions contribute to the determinants of evolution of protein families [63] and *iii) *the nature of interactions that small molecule inhibitors of protein-protein interactions engage [64]. Indeed the atomic level data in PICCOLO may assist structure based drug design efforts against protein-protein interfaces as they help identify the most critical determinants of binding.

**Figure 3:**
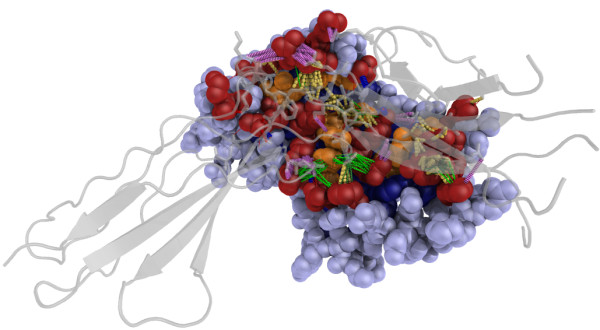
**Complex of human somatotropin and the prolactin receptor (PDB entry 1bp3)**. Residues in the interface core are shown in orange, interface periphery in dark red, non-interface exposed surface in light blue and buried protein core in dark blue. Interaction types are coloured as follows: hydrogen bonds in dark blue; water mediated hydrogen bonds in light blue; π-cation interactions in pink; ionic interactions in pink; hydrophobic contacts in yellow; and van der Waals in red. Figure prepared using PyMOL [66].

With large and complex data sets of this nature visualization tools to aid analysis are of tremendous value. PyMOL (Delano (2002)) is an open-source molecular visualization system that extends, and is extensible by the Python programming language (Van Rossum (2003)). This enables Python functions to be written that connect to the MySQL database and extract annotations from PICCOLO describing the atoms and residues that are involved in interactions, and for these to be highlighted in the PyMOL window. Furthermore, the interatomic interactions themselves can be visualized by using different colour and dash parameters to indicate different interaction types. Figure 3 shows an example of the complex of human somatotropin and the prolactin receptor (PDB entry 1bp3) indicating the jungle of molecular interactions.

While PICCOLO focuses on protein-protein interactions, parallel sister databases dealing with protein interactions with other classes of molecules have been developed within the group. BIPA [65] concerns the interactions of proteins with nucleic acids. CREDO [43] concerns the interactions of proteins with small-molecule heteratomic ligands. TIMBAL [64], is a hand-curated database comprising small molecule ligands published in the literature that are known to disrupt protein-protein interactions. TIMBAL comprises 117 small molecules, from 21 protein-protein interaction systems, 13 of which have some structural representation and can be cross-referenced to PICCOLO enabling insights into the type of molecular interactions favoured by inhibitors of protein-protein interactions. The databases were designed using highly similar interaction definitions and they share PDB residue identifiers, enabling useful comparative cross-queries to be performed.

### PICCOLO availability

PICCOLO is available through a simple web interface at the following URL http://www-cryst.bioc.cam.ac.uk/piccolo. The database comprises detailed descriptions of protein-protein interfaces at various levels of granularity for all structurally characterized complexes deposited in the PDB. Automated database updates are performed every month. In building PICCOLO our focus was on providing a resource to enable large-scale analysis of global properties of protein interfaces. To this end the entire database has been made available in the form of a MySQL dump file. However, some users may find it useful to use PICCOLO to help analyze individual systems. For example, PICCOLO could be used to identify hot-spot residues or candidates for mutagenesis based on the number and nature of intermolecular contacts. Similarly, these same properties could be used to assess determinants for evolutionary conservation. As such, a simple query interface has been provided, implemented in the popular scripting language PHP. The interface enables individual complexes to be retrieved and the details of the intermolecular interactions, described at the level of atoms, residues or polypeptide chains, to be downloaded in a range of formats.

## Conclusions

The PICCOLO database uniquely captures the details of structurally characterized protein-protein interactions at atomic level. Neither the recent efforts at achieving a systems level understanding of cellular processes, nor component-by-component reductionist approaches, offers a complete understanding of cellular processes in isolation. Rather, the reciprocal synthesis of the complementary "top-down" and "bottom-up" views of biology offers the best hope of providing true insight. Such integration requires comprehensive data describing the fundamental details of each component and its interactions. We hope PICCOLO can be useful contribution to this end.

## List of abbreviations used

ASA: Accessible Surface Area; ASU: Asymmetric Unit; nsSNP: non-synonymous Single Nucleotide Polymorphisms; NMR: Nuclear Magnetic Resonance; PDB: Protein Data Bank; PQS: Protein Quaternary Structure; PICCOLO: Protein Interaction Collection Online; PISA: Protein Interfaces, Surfaces and Assemblies.

## Authors' contributions

GRJB developed the PICCOLO software, database and front end and drafted the manuscript. AH contributed with the automated update of the resource. TLB participated in the design and coordination of the database. All authors helped to draft and have approved the final manuscript.

## Authors' information

GRJB received his Masters Degree in Molecular and Cellular Biochemistry from Balliol College, University of Oxford in 1999. He subsequently gained an MSc in Molecular Modelling and Bioinformatics from Birkbeck College, University of London before joining Inpharmatica Ltd, applying structural bioinformatics to drug discovery. After five years at Inpharmatica Richard left to undertake a PhD at the Department of Biochemistry, University of Cambridge. He is currently a postdoc in the Medicinal Informatics group of Professor Andrew Hopkins at the University of Dundee.

AH received her degree in Chemistry from Barcelona University, Spain, in 1999 and her Masters in Computational Chemistry from Bologna University, Italy, in 2000. Until 2008 she worked as a molecular modeller in De Novo Pharmaceuticals and later in UCB Celltech. Since 2007 she has been carrying out PhD studies in protein-protein interactions as drug targets in the Department of Biochemistry, University of Cambridge, UK.

TLB is in Biochemistry, University of Cambridge. Until 2009 he was Sir William Dunn Professor of Biochemistry and Head of Biological Sciences in Cambridge. His research is focused on structural biology and bioinformatics and their applications to drug discovery and medicine. Most of his work has been on multi-component protein assemblies that mediate cell regulation and relevant to cancer. Tom Blundell was member of advisory group to the Prime Minister (ACOST) in 1980s; founding Chief Executive (1994-1996), and Chair (2009 to 2013), Biotechnology and Biological Sciences Research Council; Chairman, Royal Commission on Environmental Pollution (1998 to 2005): President of UK Biosciences Federation 2001 to 2005 and Biochemical Society from 2008 to 2011. He was Non-Executive Director of Celltech (1996 to 2005), science advisor with Pfizer, UCB and SKB and co-founder Astex Therapeutics (1999) with oncology drugs in clinical trials.

## Supplementary Material

Additional file 1 **Atomic properties for each residue used in generation of PICCOLO interaction fingerprints**. Click here for file

Additional file 2 **Residue matrices used to derive the contact preference matrix **(Figure 2). The matrices describe observed interface contacts Equation 6 (Figure 2), expected pairwise frequency Equation 7 (Figure 2b) and pairwise solvent accessibility (Figure 2c). Click here for file
