# Supplementary material for: Comprehensive, atomic-level characterization of structurally characterized protein-protein interactions: the PICCOLO database
Source: BMC Bioinformatics. 2011 Jul 29;12:313. doi: 10.1186/1471-2105-12-313 (PMC3161047; doi:10.1186/1471-2105-12-313)
Supplement: Additional file 2 — Residue matrices used to derive the contact preference matrix (Figure 2). The matrices describe observed interface contacts Equation 6 (Figure 2), expected pairwise frequency Equation 7 (Figure 2b) and pairwise solvent accessibility (Figure 2c). [file 1471-2105-12-313-S2.DOC]

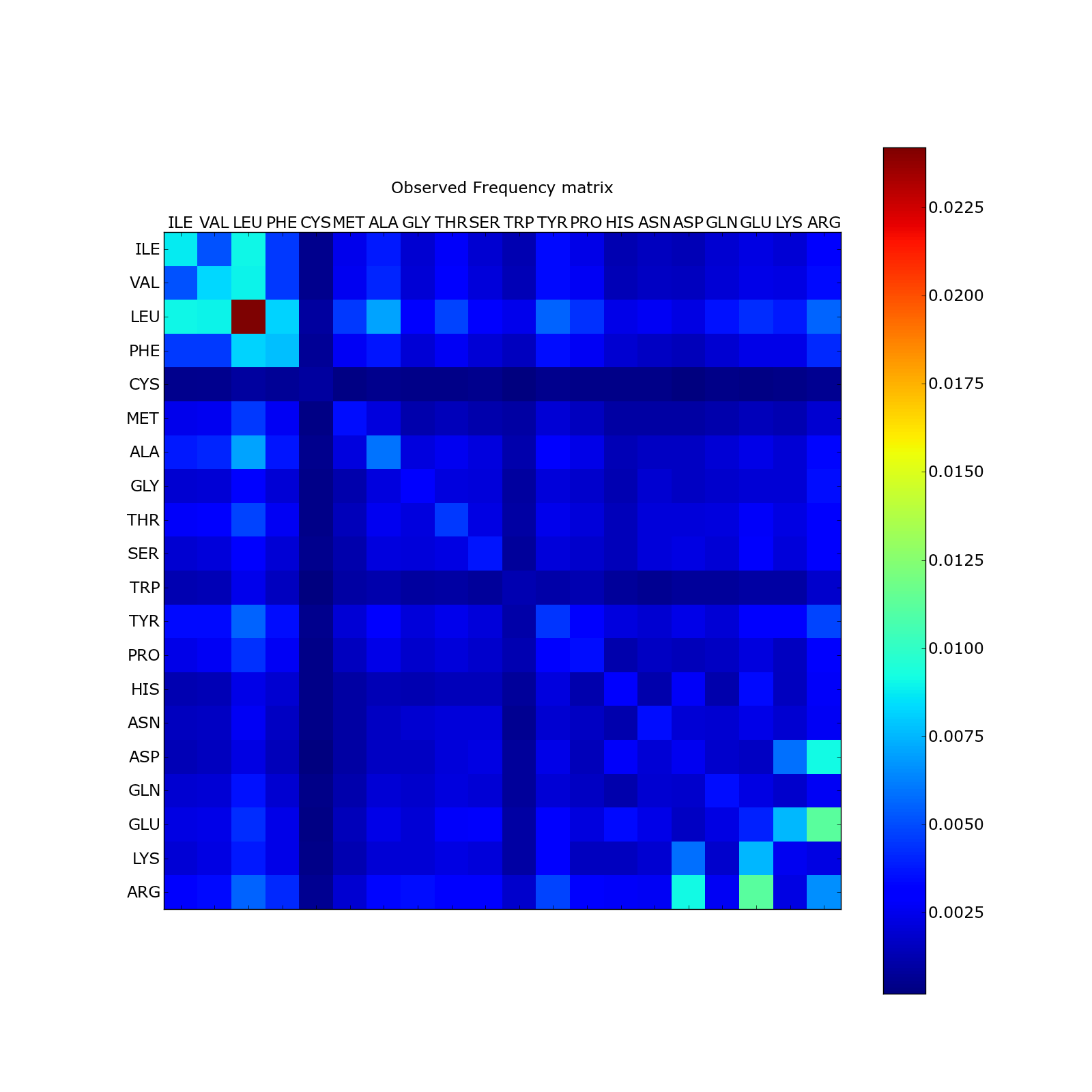


Additional file 2a: Observed contact matrix from non-redundant set of protein interfaces (Equation 6 in main text). Here, leucine-leucine contacts dominate, however this is largely due to the high abundance of leucine in general. To assess the impact of residue abundance, comparison should be made with the expected contact matrix in additional file 2b.


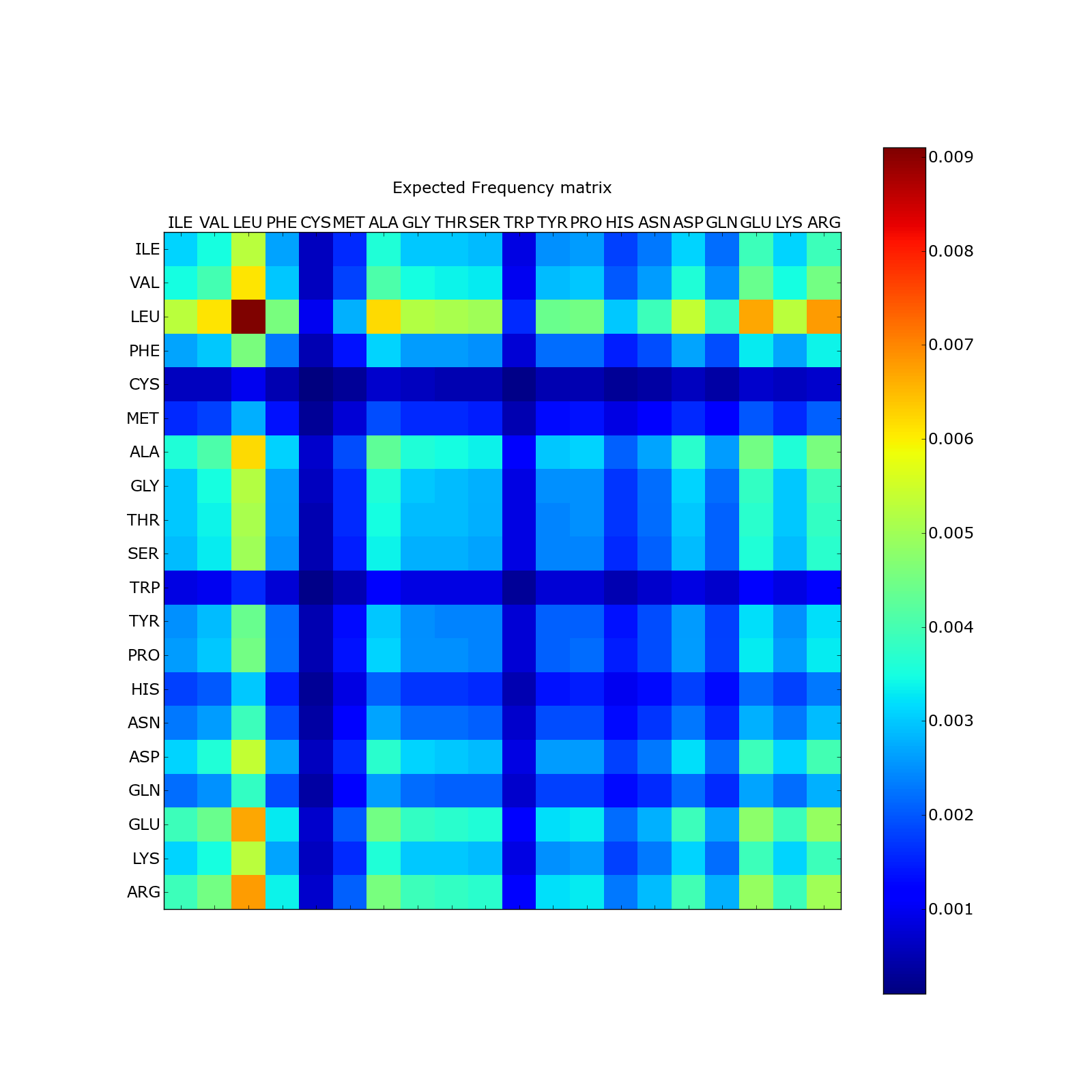


Additional file 2b: Expected contact matrix (Equation 7 in main text). This matrix does not describe observed interface contacts, rather the expected frequency of residue contacts based on the underlying frequency of the residues involved.


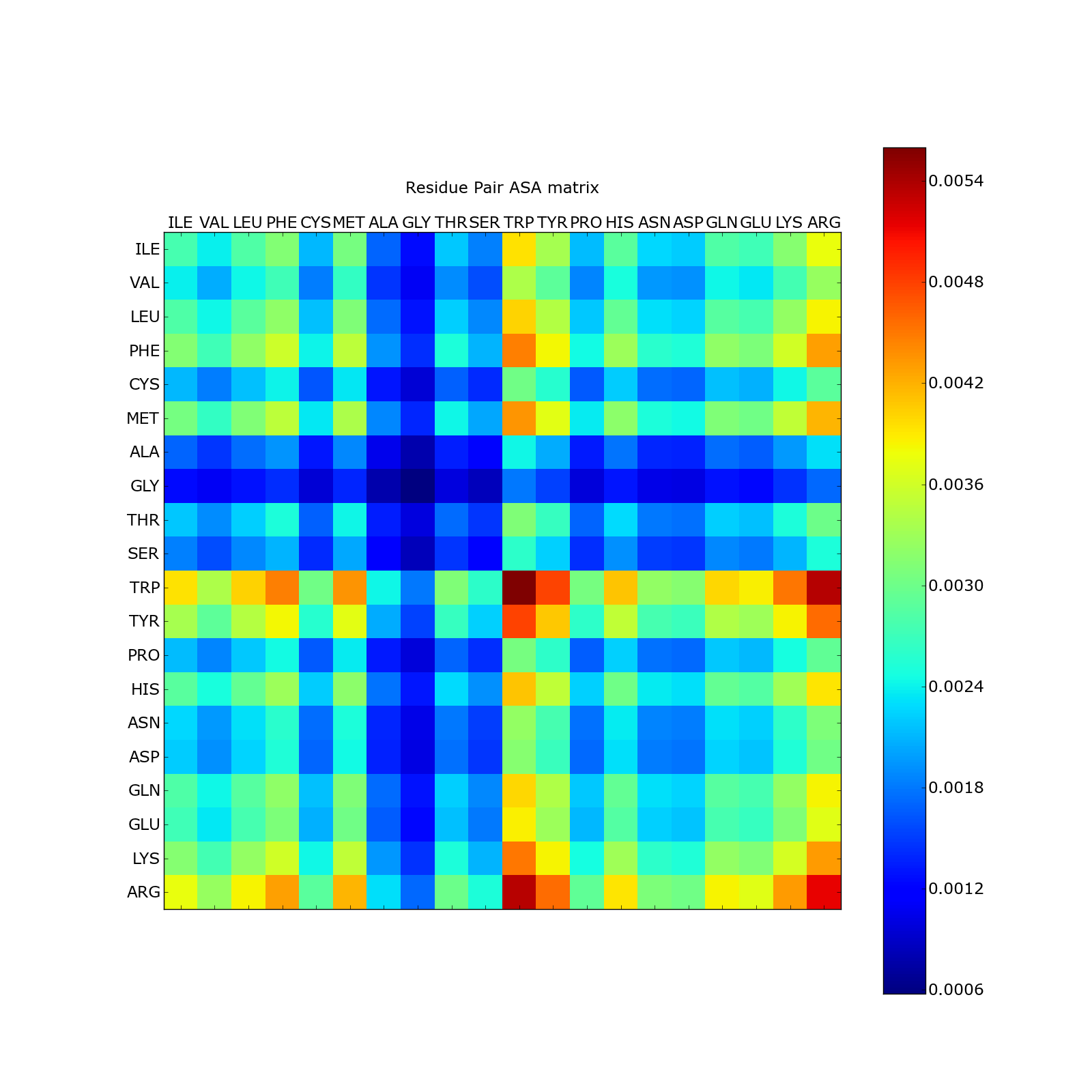


Additional file 2c: Pairwise ASA matrix. This matrix does not describe observed interface contacts, or frequencies, rather the combined pairwise solvent accessible surface area (ASA) of the residues involved. ASA data are used to normalize expected matrix in generating the final contact preference matrix (Figure 2).
